# Tobacco Use Among Middle and High School Students — United States, 2013

**Published:** 2014-11-14

**Authors:** René A. Arrazola, Linda J. Neff, Sara M. Kennedy, Enver Holder-Hayes, Christopher D. Jones

**Affiliations:** 1 Office on Smoking and Health, National Center for Chronic Disease Prevention and Health Promotion, CDC; 2 Department of Biostatistics and Epidemiology, RTI International; 3 Center for Tobacco Products, Food and Drug Administration

Tobacco use is the leading preventable cause of disease and death in the United States, and nearly all tobacco use begins during youth and young adulthood (1,2). Among U.S. youths, cigarette smoking has declined in recent years; however, the use of some other tobacco products has increased (3), and nearly half of tobacco users use two or more tobacco products (4). CDC analyzed data from the 2013 National Youth Tobacco Survey* to determine the prevalence of ever (at least once) and current (at least 1 day in the past 30 days) use of one or more of 10 tobacco products (cigarettes, cigars, hookahs, smokeless tobacco, electronic cigarettes [e-cigarettes], pipes, snus, bidis, kreteks, and dissolvable tobacco) among U.S. middle school (grades 6–8) and high school (grades 9–12) students. In 2013, 22.9% of high school students reported current use of any tobacco product, and 12.6% reported current use of two or more tobacco products; current use of combustible products (i.e., cigarettes, cigars, pipes, bidis, kreteks, and/or hookahs) was substantially greater (20.7%) than use of other types of tobacco. Also, 46.0% of high school students reported having ever tried a tobacco product, and 31.4% reported ever trying two or more tobacco products. Among middle school students, 3.1% reported current use of cigars, and 2.9% reported current use of cigarettes, with non-Hispanic black students more than twice as likely to report current use of cigars than cigarettes. Monitoring the prevalence of the use of all available tobacco products, including new and emerging products, is critical to support effective population-based interventions to prevent and reduce tobacco use among youths as part of comprehensive tobacco prevention and control programs.

The National Youth Tobacco Survey is a cross-sectional, school-based, self-administered, pencil-and-paper questionnaire administered to U.S. middle school (grades 6–8) and high school (grades 9–12) students. Information is collected on tobacco control outcome indicators to monitor the impact of comprehensive tobacco control policies and programs (5) and regulatory authorities of the Food and Drug Administration (FDA) (6). A three-stage cluster sampling procedure was used to generate a nationally representative sample of students in grades 6–12. Of 250 schools selected for the 2013 National Youth Tobacco Survey, 187 (74.8%) participated, with a sample of 18,406 (90.7%) among 20,301 eligible students†; the overall response rate was 67.8%. Participants were asked about ever and current use of cigarettes, cigars (defined as cigars, cigarillos, or little cigars), smokeless tobacco (defined as chewing tobacco, snuff, or dip), pipes, bidis, kreteks, hookah, snus, dissolvable tobacco, and e-cigarettes. Ever use was defined as ever trying a product, and current use was defined as using a product on 1 or more days during the past 30 days. For both ever use and current use, any tobacco use was defined as reporting the use of one or more tobacco products; use of two or more tobacco products was defined as reporting the use of two or more tobacco products in the specified time, current (in the past 30 days) or ever. Combustible tobacco was defined as cigarettes, cigars, pipes, bidis, kreteks, and/or hookahs. Noncombustible tobacco was defined as smokeless tobacco, snus, and/or dissolvable tobacco. A separate category was created for e-cigarette use. Data were adjusted for nonresponse and weighted to provide national prevalence estimates with 95% confidence intervals; statistically significant (p<0.05) differences between population subgroups were assessed using a t-test. Estimates for ever and current use are presented for each type of product, for any tobacco use, and for the use of two or more tobacco products by selected demographics for each school level (middle and high).

In 2013, 22.9% of high school students reported current use of a tobacco product, including 12.6% who reported current use of two or more tobacco products. Among all high school students, cigarettes (12.7%) and cigars (11.9%) were the most commonly reported tobacco products currently used, followed by smokeless tobacco (5.7%), hookahs (5.2%), e-cigarettes (4.5%), pipes (4.1%), snus (1.8%), kreteks (0.8%), bidis (0.6%), and dissolvable tobacco (0.4%) (Table 1). Among high school students who identified as non-Hispanic white or Hispanic, cigarettes were the product most commonly used, whereas cigar use was more common for all other race/ethnicities. Cigar use among non-Hispanic black students was nearly 50% higher than cigarette use. Younger children are less likely to try tobacco than older children with the proportions of current any tobacco users and current users of two or more tobacco products being lower among middle school students (6.5% and 2.9%, respectively) than high school students (22.9% and 12.6%, respectively). Cigars (3.1%) and cigarettes (2.9%) were the most commonly reported tobacco products currently used by middle school students, followed by pipes (1.9%); smokeless tobacco (1.4%); e-cigarettes and hookahs (1.1%); and bidis, kreteks, and snus (0.4%). The proportions of ever users of any tobacco product and ever users of two or more tobacco products were higher among high school (46.0% and 31.4%, respectively) than middle school (17.7% and 9.4%, respectively) students (Table 2).

Combustible tobacco products were the most commonly used form of tobacco among both current and ever tobacco users (Figure). Among high school students, 20.7% currently used combustible products (13.5% combustible only; 3.4% combustible and noncombustible only; 2.7% combustible and e-cigarettes only; and 1.1% combustible, noncombustible, and e-cigarettes). Of all middle school students, 5.4% currently used combustible products (4.0% combustible only; 0.8% combustible and noncombustible only; 0.4% combustible and e-cigarettes only; and 0.2% combustible, noncombustible, and e-cigarettes). Current use of only e-cigarettes was 0.6% among high school students and 0.4% among middle school students.

## Discussion

In 2013, more than one in five high school students (22.9%) and more than one in 20 middle school students (6.5%) reported using a tobacco product on 1 or more days during the past 30 days. In addition, nearly half of high school students (46.0%) and almost one in five of middle school students (17.7%) had ever used tobacco. These findings indicate that continued efforts are needed to monitor and prevent the use of all forms of tobacco use among youths.

Combustible tobacco use remains the most common type of tobacco use and causes most tobacco-related disease and death in the United States (1). Nine out of 10 high school current and ever tobacco users used a combustible tobacco product (Figure). There was lower use of only noncombustible tobacco products or only e-cigarettes among both current and ever tobacco users. However, noncombustible products also pose health risks (7). Smokeless tobacco is not a safe alternative to combustible tobacco because it causes cancer and nicotine addiction (7). In addition, although the long-term impact of e-cigarette use on public health overall remains uncertain, the 2014 Surgeon General’s report found that nicotine use can have adverse effects on adolescent brain development; therefore, nicotine use by youths in any form (whether combustible, smokeless, or electronic) is unsafe (1).

Most youths who currently use tobacco believe that they will be able to stop using tobacco in the near future; unfortunately, however, many continue use well into adulthood (2). Youths who report use of multiple tobacco products are at higher risk for developing nicotine dependence; about two thirds (62.9%) of youths who use more than one tobacco product report tobacco dependence symptoms, compared with 36.0% of those who use one tobacco product (8). Thus, youths who use multiple tobacco products might be more likely to continue using tobacco into adulthood. Comprehensive youth tobacco prevention programs that prevent initiation of all types of tobacco products are critical to protect youths from tobacco use and nicotine dependence.

The findings in this report are subject to at least five limitations. First, data were only collected from youths who attended either public or private schools and might not be generalizable to all middle and high school-aged youths. Second, data were self-reported; thus, the findings are subject to recall and/or response bias. Third, current and ever tobacco use were estimated by including students who responded to using at least one of the 10 tobacco products included in the survey but might have had missing responses to any of the other nine tobacco products; missing responses were considered as nonuse, which might have resulted in conservative estimates. Fourth, nonresponse bias might have affected the results because the survey response rate was only 67.8%. Finally, estimates might differ from those derived from other nationally representative youth surveillance systems, in part because of differences in survey methods, survey type and topic, and age and setting of the target population. However, overall prevalence estimates are similar across the various youth surveys (2).

Although substantial progress has been made in decreasing cigarette use among youths (2), overall tobacco use is still high, with one in five high school students currently using tobacco and nearly half reporting they have ever used a tobacco product. Ever using a tobacco product is a concern because even one-time use of tobacco is associated with increased long-term risks for becoming a regular user (2). In April 2014, FDA issued a proposed rule to extend its jurisdiction over the manufacture, marketing, and distribution of tobacco products not currently regulated by FDA, which includes cigars, e-cigarettes, pipes, and hookahs (9). FDA is reviewing the comments received on this proposed rule. Full implementation of comprehensive tobacco control programs at CDC-recommended funding levels would be expected to result in further reductions in tobacco use and changes in social norms regarding the acceptability of tobacco use among U.S. youths (1,2,10). Additionally, considering how trends in tobacco product use and tobacco marketing changes, rigorous surveillance of all available forms of tobacco use by youths, particularly emerging products such as e-cigarettes, is essential. Rigorous surveillance of the use of all types of tobacco will inform enhanced prevention efforts that could protect the estimated 5.6 million youths in the United States currently projected to die prematurely from a smoking-related disease (1).

What is already known on this topic?

Nearly all tobacco use begins during youth and young adulthood. Among U.S. youths, declines have occurred in the prevalence of cigarette smoking in recent years; however, the use of some other tobacco products has increased, and nearly half of tobacco users use two or more tobacco products.

What is added by this report?

In 2013, 22.9% of high school students reported current use (use on 1 or more days in the past 30 days) of any tobacco product, and 12.6% reported current use of two or more tobacco products. Forty-six percent of high school students reported having ever tried a tobacco product, and 31.4% reported ever trying two or more tobacco products. The most common types of tobacco products currently used by high school students were combustibles (i.e., cigarettes, cigars, pipes, bidis, kreteks, and/or hookahs) (20.7%). Among middle school students, 3.1% reported current use of cigars, and 2.9% reported current use of cigarettes.

What are the implications for public health practice?

Despite recent reductions in tobacco use, the one in five high school students who reported current use of tobacco and the almost half who reported ever using a tobacco product remain at risk for nicotine dependence and the adverse health consequences of tobacco use. Considering how trends in tobacco product use and tobacco marketing changes, rigorous surveillance of all available forms of tobacco use by youths, particularly use of emerging products such as e-cigarettes, is essential.

## Figures and Tables

**FIGURE:**
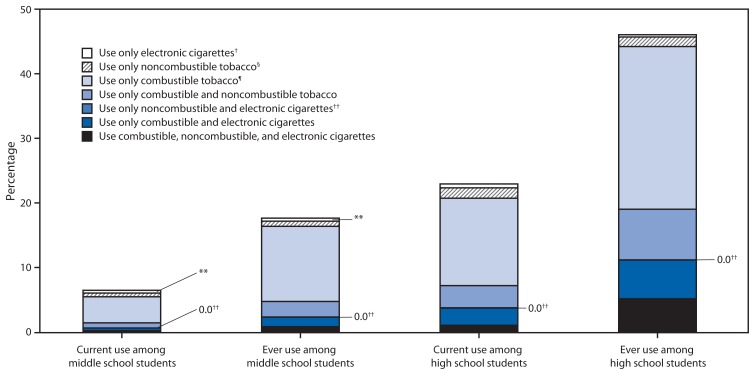
Tobacco use* among middle and high school students — National Youth Tobacco Survey, United States, 2013 ^*^ Tobacco is use of cigarettes, cigars, smokeless tobacco, tobacco pipes, bidis, kreteks, hookah, snus, dissolvable tobacco, and/or electronic cigarettes. ^†^ Only electronic cigarette use is exclusive use of only electronic cigarettes. It does not include use of any other product. ^§^ Only noncombustible tobacco use is exclusive use of only smokeless tobacco, snus, and/or dissolvable tobacco. It does not include use of combustible products or electronic cigarettes. ^¶^ Only combustible tobacco use is exclusive use of only cigarettes, cigars, pipes, bidis, kreteks, and/or hookah. It does not include use of noncombustible products or electronic cigarettes. ^**^ Data statistically unstable because relative standard error is >0.3. ^††^ Percentages for only noncombustible and electronic cigarettes are minimal but are indicated between only combustible and noncombustible use and only combustible and electronic cigarette use. Data are statistically unstable because relative standard error is >0.3.

**TABLE 1:** Percentage of current use* of tobacco, by product, school level, sex, and race/ethnicity — National Youth Tobacco Survey, United States, 2013

			Sex				Race/Ethnicity							
			
	Total		Female		Male		White, non-Hispanic		Black, non-Hispanic		Hispanic		Other race, non-Hispanic	
							
Tobacco product	%	(95% CI)	%	(95% CI)	%	(95% CI)	%	(95% CI)	%	(95% CI)	%	(95% CI)	%	(95% CI)
**Middle school students**														
Cigarettes	2.9	(2.3–3.6)	2.8	(2.1–3.8)	3.0	(2.3–3.8)	2.6	(1.9–3.6)	1.7	(1.1–2.8)	5.1	(3.9–6.7)	—†	—
Cigars	3.1	(2.4–3.9)	2.9	(2.1–4.0)	3.3	(2.6–4.2)	2.2	(1.4–3.3)	4.5	(3.0–6.7)	4.7	(3.7–6.0)	—	—
Smokeless tobacco	1.4	(0.9–2.0)	0.8	(0.6–1.2)	1.9	(1.2–2.8)	1.4	(0.8–2.5)	—	—	1.8	(1.2–2.8)	—	—
Pipes	1.9	(1.4–2.4)	2.2	(1.5–3.2)	1.6	(1.1–2.2)	—	—	1.7	(1.1–2.7)	4.3	(3.2–5.7)	—	—
Bidis	0.4	(0.3–0.6)	0.4	(0.2–0.6)	0.5	(0.3–0.8)	—	—	—	—	—	—	—	—
Kreteks	0.4	(0.3–0.5)	—	—	0.5	(0.3–0.8)	0.2	(0.1–0.5)	—	—	—	—	—	—
Hookah	1.1	(0.8–1.5)	1.3	(0.8–1.9)	0.9	(0.6–1.4)	0.7	(0.4–1.2)	—	—	2.4	(1.5–3.6)	—	—
Snus	0.4	(0.3–0.6)	—	—	0.7	(0.4–1.0)	0.3	(0.2–0.6)	—	—	—	—	—	—
Dissolvable tobacco	—	—	—	—	—	—	—	—	—	—	—	—	—	—
Electronic cigarettes	1.1	(0.8–1.5)	0.9	(0.6–1.4)	1.4	(1.0–1.9)	0.9	(0.6–1.4)	—	—	1.8	(1.1–2.7)	—	—
**Any tobacco product use**§	**6.5**	**(5.4–7.8)**	**6.5**	**(5.3–7.9)**	**6.5**	**(5.3–8.0)**	**5.6**	**(4.2–7.3)**	**6.8**	**(5.4–8.6)**	**9.7**	**(8.0–11.7)**	**3.5**	**(2.0–6.0)**
**≥2 tobacco product use**¶	**2.9**	**(2.3–3.7)**	**2.6**	**(1.8–3.8)**	**3.2**	**(2.5–4.0)**	**2.2**	**(1.6–3.1)**	**2.3**	**(1.4–3.9)**	**5.5**	**(4.4–6.9)**	**—**	**—**
**High school students**														
Cigarettes	12.7	(11.3–14.2)	11.2	(9.8–12.9)	14.1	(12.3–16.1)	14.0	(12.2–16.0)	9.0	(7.0–11.5)	13.4	(11.1–16.2)	7.6	(4.9–11.6)
Cigars	11.9	(10.8–13.2)	8.3	(6.9–9.8)	15.4	(13.9–17.0)	11.4	(10.2–12.8)	14.7	(12.3–17.4)	12.1	(10.2–14.3)	8.5	(5.0–14.0)
Smokeless tobacco	5.7	(4.5–7.2)	1.7	(1.2–2.3)	9.6	(7.6–12.0)	7.5	(5.6–9.8)	2.4	(1.5–3.9)	4.0	(3.1–5.0)	—	—
Pipes	4.1	(3.5–4.9)	3.3	(2.7–4.0)	5.0	(4.1–6.0)	3.7	(2.9–4.7)	3.5	(2.6–4.7)	6.5	(5.5–7.7)	—	—
Bidis	0.6	(0.5–0.9)	0.5	(0.3–0.8)	0.8	(0.6–1.2)	0.6	(0.4–0.9)	—	—	0.7	(0.4–1.2)	—	—
Kreteks	0.8	(0.6–1.1)	0.5	(0.3–0.8)	1.2	(0.8–1.6)	1.0	(0.7–1.4)	—	—	0.7	(0.4–1.2)	—	—
Hookah	5.2	(4.6–6.0)	4.8	(4.1–5.7)	5.6	(4.7–6.7)	5.3	(4.6–6.2)	2.4	(1.6–3.4)	7.1	(5.8–8.6)	6.4	(3.6–11.1)
Snus	1.8	(1.4–2.3)	0.9	(0.6–1.4)	2.7	(2.1–3.5)	2.4	(1.8–3.2)	—	—	1.3	(0.9–2.0)	—	—
Dissolvable tobacco	0.4	(0.3–0.6)	—	—	0.6	(0.4–0.9)	0.3	(0.2–0.6)	—	—	0.5	(0.3–0.9)	—	—
Electronic cigarettes	4.5	(3.8–5.3)	3.5	(2.8–4.3)	5.5	(4.5–6.8)	4.8	(3.8–6.1)	2.7	(1.9–3.9)	5.3	(4.2–6.6)	4.0	(2.3–6.8)
**Any tobacco product use**§	**22.9**	**(21.1–24.8)**	**18.5**	**(16.8–20.2)**	**27.2**	**(24.6–30.0)**	**24.0**	**(21.6–26.5)**	**21.0**	**(18.3–23.9)**	**23.9**	**(21.6–26.4)**	**14.8**	**(10.2–21.0)**
**≥2 tobacco product use**¶	**12.6**	**(11.5–13.8)**	**8.7**	**(7.7–9.8)**	**16.4**	**(14.7–18.2)**	**14.0**	**(12.5–15.6)**	**8.2**	**(6.7–10.1)**	**13.5**	**(11.7–15.6)**	**8.7**	**(5.5–13.6)**

**Abbreviation:** CI = confidence interval.

* Current use of cigarettes was determined by asking, “During the past 30 days, on how many days did you smoke cigarettes?”; Current use of cigars was determined by asking, “During the past 30 days, on how many days did you smoke cigars, cigarillos, or little cigars?”; Current use of smokeless tobacco was determined by asking, “During the past 30 days, on how many days did you use chewing tobacco, snuff, or dip?”; Current use of pipe was determined by asking, “During the past 30 days, on how many days did you smoke tobacco in a pipe?”; Current use of bidis, kreteks, hookah, snus, dissolvable tobacco, and electronic cigarettes were determined by asking, “During the past 30 days, which of the following products have you used on at least 1 day?”. Any respondent who responded affirmatively to any of these questions was considered a current user of tobacco.

† Data are statistically unstable because sample size <50 or relative standard error >0.3.

§ Any tobacco product use is current use of cigarettes, cigars, smokeless tobacco, tobacco pipes, bidis, kreteks, hookah, snus, dissolvable tobacco, and/or electronic cigarettes.

¶ Two or more tobacco product use is current use of products from two or more of the following categories: cigarettes, cigars, smokeless tobacco, tobacco pipes, bidis, kreteks, hookah, snus, dissolvable tobacco, and/or electronic cigarettes.

**TABLE 2:** Percentage of ever use* of tobacco, by product, school level, sex, and race/ethnicity — National Youth Tobacco Survey, United States, 2013

			Sex				Race/Ethnicity							
			
	Total		Female		Male		White, non-Hispanic		Black, non-Hispanic		Hispanic		Other race, non-Hispanic	
							
Tobacco product	%	(95% CI)	%	(95% CI)	%	(95% CI)	%	(95% CI)	%	(95% CI)	%	(95% CI)	%	(95% CI)
**Middle school students**														
Cigarettes	12.7	(10.9–14.8)	12.0	(10.1–14.1)	13.5	(11.5–15.7)	11.0	(8.9–13.4)	13.2	(9.7–17.8)	18.9	(15.8–22.4)	9.0	(6.6–12.2)
Cigars	8.9	(7.5–10.5)	7.5	(6.2–9.1)	10.2	(8.7–12.1)	6.8	(5.6–8.3)	13.6	(9.4–19.3)	12.4	(10.3–15.0)	6.4	(4.1–9.9)
Smokeless tobacco	3.6	(2.8–4.6)	1.9	(1.3–2.7)	5.2	(4.0–6.8)	3.8	(2.7–5.3)	—†	—	4.6	(3.3–6.2)	2.6	(1.4–4.6)
Pipes	4.1	(3.3–5.0)	4.1	(3.1–5.4)	4.0	(3.2–5.1)	3.4	(2.6–4.3)	3.5	(2.3–5.3)	7.1	(5.3–9.4)	—	—
Bidis	0.8	(0.6–1.1)	0.6	(0.4–0.9)	1.1	(0.7–1.6)	0.6	(0.3–0.9)	—	—	1.5	(1.0–2.3)	—	—
Kreteks	0.6	(0.5–0.8)	0.5	(0.3–0.8)	0.8	(0.5–1.3)	0.6	(0.4–1.0)	—	—	1.0	(0.5–1.7)	—	—
Hookahs	3.0	(2.4–3.7)	3.0	(2.2–4.1)	2.9	(2.3–3.8)	2.4	(1.8–3.2)	—	—	5.9	(4.5–7.8)	—	—
Snus	1.3	(1.0–1.7)	0.9	(0.6–1.5)	1.7	(1.2–2.3)	1.6	(1.1–2.3)	—	—	1.7	(1.0–2.7)	—	—
Dissolvable tobacco	0.5	(0.3–0.8)	—	—	0.8	(0.5–1.2)	—	—	—	—	—	—	—	—
Electronic cigarettes	3.0	(2.5–3.5)	2.8	(2.3–3.5)	3.1	(2.6–3.9)	3.0	(2.4–3.7)	2.7	(1.9–3.7)	3.9	(2.9–5.2)	—	—
**Any tobacco product use**§	**17.7**	**(15.6–19.9)**	**16.2**	**(14.1–18.6)**	**19.0**	**(16.7–21.6)**	**15.4**	**(13.0–18.0)**	**20.3**	**(15.9–25.5)**	**23.9**	**(21.0–27.2)**	**11.7**	**(8.5–15.8)**
**≥2 tobacco product use**¶	**9.4**	**(7.9–11.1)**	**8.5**	**(7.0–10.4)**	**10.2**	**(8.6–12.1)**	**8.0**	**(6.4–9.9)**	**9.5**	**(6.5–13.7)**	**14.9**	**(12.5–17.6)**	**5.9**	**(3.8–9.2)**
**High school students**														
Cigarettes	34.7	(31.8–37.7)	33.0	(29.8–36.2)	36.3	(33.0–39.8)	33.9	(30.2–37.8)	33.8	(29.3–38.7)	40.2	(36.4–44.1)	24.6	(18.3–32.1)
Cigars	30.5	(28.4–32.8)	24.5	(22.3–26.8)	36.4	(33.6–39.3)	30.4	(28.0–32.9)	34.9	(30.3–39.7)	30.4	(27.3–33.7)	20.3	(13.5–29.3)
Smokeless tobacco	13.4	(11.3–15.7)	5.6	(4.3–7.3)	20.8	(17.7–24.3)	17.5	(14.7–20.7)	6.6	(5.0–8.6)	8.7	(7.1–10.5)	5.8	(3.6–9.3)
Pipes	9.7	(8.6–11.0)	7.4	(6.3–8.6)	12.0	(10.4–13.7)	10.5	(9.1–12.0)	6.1	(4.9–7.6)	11.1	(9.4–13.1)	6.3	(3.7–10.5)
Bidis	2.6	(2.1–3.2)	1.4	(1.0–1.9)	3.8	(3.1–4.6)	2.8	(2.2–3.7)	2.1	(1.5–2.9)	2.8	(2.2–3.5)	—	—
Kreteks	2.8	(2.3–3.4)	1.4	(1.0–1.9)	4.2	(3.4–5.1)	3.7	(2.9–4.5)	1.6	(1.0–2.4)	1.7	(1.2–2.5)	—	—
Hookahs	14.3	(12.7–16.0)	13.5	(11.7–15.4)	15.1	(13.2–17.2)	15.5	(14.0–17.3)	7.4	(5.1–10.5)	17.4	(14.9–20.2)	11.0	(7.1–16.7)
Snus	6.2	(5.1–7.5)	2.9	(2.3–3.7)	9.4	(7.5–11.6)	8.5	(7.0–10.3)	1.6	(0.9–2.8)	4.3	(3.3–5.6)	—	—
Dissolvable tobacco	1.0	(0.8–1.3)	0.4	(0.3–0.7)	1.6	(1.2–2.2)	1.1	(0.8–1.5)	0.9	(0.6–1.6)	1.0	(0.6–1.6)	—	—
Electronic cigarettes	11.9	(10.5–13.5)	9.9	(8.3–11.7)	13.8	(12.1–15.7)	14.7	(12.8–16.9)	4.9	(3.6–6.5)	10.4	(8.6–12.5)	8.3	(5.3–12.7)
**Any tobacco product use**§	**46.0**	**(43.4–48.7)**	**41.8**	**(38.8–44.8)**	**50.1**	**(47.0–53.2)**	**45.2**	**(41.8–48.7)**	**48.3**	**(43.6–53.1)**	**49.9**	**(47.2–52.5)**	**33.4**	**(25.2–42.8)**
**≥2 tobacco product use**¶	**31.4**	**(29.0–33.9)**	**26.4**	**(24.1–28.7)**	**36.2**	**(33.2–39.3)**	**33.3**	**(30.2–36.5)**	**26.6**	**(23.9–29.5)**	**33.5**	**(30.4–36.8)**	**18.8**	**(13.1–26.1)**

* Ever use of cigarettes was determined by asking, “Have you ever tried cigarette smoking, even one or two puffs?”; Ever use of cigars was determined by asking, “Have you ever tried smoking cigars, cigarillos, or little cigars, such as Black and Milds, Swisher Sweets, Dutch Masters, White Owl, or Phillies Blunts, even one or two puffs?; Ever use of smokeless tobacco was determined by asking, “Have you ever used chewing tobacco, snuff, or dip, such as Redman, Levi Garrett, Beechnut, Skoal, Skoal Bandits, or Copenhagen, even just a small amount?”; Ever use of pipe was determined by asking, “Have you ever tried smoking tobacco in a pipe, even one or two puffs?”; Ever use of bidis, kreteks, hookah, snus, dissolvable tobacco, and electronic cigarettes were determined by asking, “Which of the following tobacco products have you ever tried, even just one time: bidis, kreteks, hookah, snus, dissolvable tobacco, and electronic cigarettes?”. Any respondent who answered affirmatively was considered to have ever used the product.

† Data are statistically unstable because sample size <50 or relative standard error >0.3.

§ Any tobacco product use is current use of cigarettes, cigars, smokeless tobacco, tobacco pipes, bidis, kreteks, hookah, snus, dissolvable tobacco, and/or electronic cigarettes.

¶ Two or more tobacco product use is current use of products from two or more of the following categories: cigarettes, cigars, smokeless tobacco, tobacco pipes, bidis, kreteks, hookah, snus, dissolvable tobacco, and/or electronic cigarettes.

## Footnotes

* Additional information available at http://www.cdc.gov/tobacco/data_statistics/surveys/nyts/index.htm.

† Additional information available at http://www.cdc.gov/tobacco/data_statistics/surveys/nyts/index.htm.
